# Identification of copy number variations among fetuses with isolated ultrasound soft markers in pregnant women not of advanced maternal age

**DOI:** 10.1186/s13023-024-03066-4

**Published:** 2024-02-10

**Authors:** Yunyun Liu, Sha Liu, Jianlong Liu, Ting Bai, Xiaosha Jing, Cechuan Deng, Tianyu Xia, Jing Cheng, Lingling Xing, Xiang Wei, Yuan Luo, Quanfang Zhou, Dan Xie, Yueyue Xiong, Ling Liu, Qian Zhu, Hongqian Liu

**Affiliations:** 1 Department of Medical Genetics, West China Second University Hospital, Sichuan University, Chengdu, China; 2 Department of Obstetrics and Gynecology, West China Second University Hospital, Sichuan University, Chengdu, China; 3 Key Laboratory of Birth Defects and Related Diseases of Women and Children (Sichuan University), Ministry of Education, Chengdu, China; 4 Prenatal Diagnostic Center, The Third Affiliated Hospital of Zhengzhou University, Zhengzhou, China

**Keywords:** Copy number variants, Ultrasound soft markers, Noninvasive prenatal screening, Positive predictive value

## Abstract

**Background:**

Pathogenic (P) copy number variants (CNVs) may be associated with second-trimester ultrasound soft markers (USMs), and noninvasive prenatal screening (NIPS) can enable interrogate the entire fetal genome to screening of fetal CNVs. This study evaluated the clinical application of NIPS for detecting CNVs among fetuses with USMs in pregnant women not of advanced maternal age (AMA).

**Results:**

Fetal aneuploidies and CNVs were identified in 6647 pregnant women using the Berry Genomics NIPS algorithm.Those with positive NIPS results underwent amniocentesis for prenatal diagnosis. The NIPS and prenatal diagnosis results were analyzed and compared among different USMs. A total of 96 pregnancies were scored positive for fetal chromosome anomalies, comprising 37 aneuploidies and 59 CNVs. Positive predictive values (PPVs) for trisomy 21, trisomy 18, trisomy 13, and sex chromosome aneuploidies were 66.67%, 80.00%, 0%, and 30.43%, respectively. NIPS sensitivity for aneuploidies was 100%. For CNVs, the PPVs were calculated as 35.59% and false positive rate of 0.57%. There were six P CNVs, two successfully identified by NIPS and four missed, of which three were below the NIPS resolution limit and one false negative. The incidence of aneuploidies was significantly higher in fetuses with absent or hypoplastic nasal bone, while that of P CNVs was significantly higher in fetuses with aberrant right subclavian artery (ARSA), compared with other groups.

**Conclusions:**

NIPS yielded a moderate PPV for CNVs in non-AMA pregnant women with fetal USM. However, NIPS showed limited ability in identifying P CNVs. Positive NIPS results for CNVs emphasize the need for further prenatal diagnosis. We do not recommend the use of NIPS for CNVs screening in non-AMA pregnant women with fetal USM, especially in fetuses with ARSA.

**Supplementary Information:**

The online version contains supplementary material available at 10.1186/s13023-024-03066-4.

## Introduction

Ultrasound soft markers (USMs) refer to minor sonographic findings often transient and distinct from fetal structural malformations that suggest an increased risk of underlying fetal aneuploidy [1, 2]. The association between trisomy 21 (T21) and several USMs has been examined, including echogenic intracardiac focus (EIF), absent or hypoplastic nasal bone, mild pyelectasis, echogenic bowel, single umbilical artery (SUA), and aberrant right subclavian artery (ARSA) [3–8]. Furthermore, the association between trisomy 18 (T18) and choroid plexus cysts (CPCs) has been elucidated; studies have shown that the prevalence of isolated CPCs in fetuses with T18 is 5.8–6.7% [9, 10], and the likelihood ratio (LR) associated with isolated CPCs for T18 ranges from 7.1 to 13.8 [10, 11]. Therefore, CPCs are generally considered to be an USM for an increased risk of T18. One study has reported an increase in the detection rate of malformations by 4% due to USMs findings [12].

Copy number analysis is a well-known first-tier approach for the prenatal diagnosis of fetuses with structural anomalies [13] and has also recently been used for the genetic etiological diagnoses of fetuses with USM. Previous studies have suggested an association between pathogenic (P) copy-number variants (CNVs) and second-trimester USMs, such as EIF and echogenic bowel associated with 16p13.11 recurrent microdeletion, mild ventriculomegaly and CPCs associated with 1q21.1 recurrent microduplication, echogenic bowel associated with the 17q12 recurrent region, and ARSA associated with 22q11.2 deletions [8, 14, 15]. Chromosomal microarray (CMA) and next-generation sequencing-based CNV analysis (Copy number variant sequence, CNV-seq) are used to detect CNVs in fetuses with USMs via invasive prenatal diagnosis. However, Lee et al. [16] showed that maternal anxiety and unnecessary amniocentesis are associated with the detection and interpretation of USMs.

Noninvasive prenatal screening (NIPS) is highly accurate in detecting common aneuploidies (T21, T18, and trisomy 13 (T13)) and sex chromosome aneuploidies (SCAs) [17]. The ability of genome-wide NIPS to detect CNVs has also received much attention in recent years, which is possible to screen fetal CNVs because of this technology can interrogate the entire fetal genome. Currently, the detection of genome-wide CNVs is performed as part of the NIPS. The prevalence of 22q11.2 deletion syndrome ranges from 1 in 990 to 1 in 2148, making it the most common prenatally-identified pathogenic CNV (P CNV). Meanwhile, the positive predictive values (PPVs) of NIPS for 22q11.2 deletion syndrome ranges from 18.5% to 100%, and is significantly higher in fetuses with abnormal ultrasound findings [17]. And based on a cost-effectiveness analysis, the American College of Medical Genetics and Genomics (ACMG) has recommended that NIPS should be offered to all patients as a prenatal method for screening the 22q11.2 deletion syndrome [18].

Currently, China has clear clinical guidelines on prenatal diagnosis requirements for pregnant women of advanced maternal age (AMA) and fetuses with structural abnormalities. AMA is associated with chromosomal abnormalities and adverse pregnancy outcomes, especially in cases with ultrasound abnormalities such as USM and structural abnormalities [19], Moreover, a study shows that increasing maternal age is associated with increasing risks of de novo or non-complex CNV, which are associated with neonatal developmental delays and intellectual disabilities [20]; however, there are no corresponding clinical guidelines for non-AMA pregnant women carrying fetuses with USM. Therefore, we conducted this study and evaluated the detection of CNVs using NIPS in non-AMA pregnant women with fetal USM. Our objectives were to assess the clinical PPVs of NIPS-based common aneuploidies and CNVs detection in fetuses with USMs, and determine the PPVs of NIPS for common aneuploidies and CNVs among different USM groups. We also explored the prevalence rates of pathogenic chromosomal aberrations in different soft marker groups to improve clinical genetic counseling.

## Methods

### Study design and participants

From January 2020 to December 2022, pregnancy cases with isolated fetal soft markers identified in ultrasonographs of the second-trimester ultrasound examination, conducted in the Department of Diagnostic Ultrasound of West China Second University Hospital, Sichuan University, and performed NIPS in Department of Medical Genetics were included in the analysis.

The exclusion criteria were as follows: (1) pregnant women with AMA (aged over 35 years at the expected date of confinement), (2) pregnant women with failed NIPS tests, including sequencing failure, and high cell free DNA concentration (> 0.6 ng/µL), (3) no clinical pregnancy outcome, including high-risk NIPS cases who declined amniocentesis and further examination, or termination of pregnancy with unclear prenatal diagnosis result.

The study was approved by the Institutional Ethics Committee of Sichuan University, and all methods were performed following the relevant guidelines and regulations.

### Ultrasound examination

First-trimester ultrasound examinations for nuchal translucency and detailed second-trimester fetal anomaly scans were performed by two experienced fetal sonographers (Voluson E8; GE Medical Systems, Zipf, Austria) [2, 14]. Fetuses with nuchal translucency of ≥ 3.0 mm, or structural abnormalities were excluded, while those with the following eight types of isolated soft markers were included in this study: EIF, mild ventriculomegaly (> 10 mm and < 12 mm), CPCs, echogenic bowel, mild pyelectasis (dilatation of the renal pelvis ≥ 4 mm), single umbilical artery (SUA), absent or hypoplastic nasal bone (absent or < 2.5 mm), and ARSA.

In twin pregnancies, chorionic and amniotic cysts in the twins were detected during the first trimester, and the occurrence of vanishing twin syndrome (VTS) should be monitored. The presence of complex twin syndrome (i.e., miscarriage or death of one of the twins, combined structural malformations in one of the twins, selective fetal growth restriction (sFGR) in twins, and/or twin-to-twin transfusion syndrome) was determined during the second trimester, and only one or two fetuses with USM were included in the study.

### Pre-test NIPS genetic counseling

Pre-test genetic counseling for NIPS was performed by trained clinical geneticists after a nuchal translucency scan; written informed consent was obtained from all the pregnant women who agreed to undergo NIPS. Genetic counseling was done with the pregnant women, and their families if desired.

Pre-test genetic counseling informed pregnant women about the examination scope, detection rate of target diseases, examination gestational age, accuracy, limitations of NIPS, maternal serum screening, and prenatal diagnosis. The pregnant women were informed of the detection rate, sensitivity, specificity, and PPV of NIPS for T21, T18, T13. Women who were pregnant with twins or VTS were also informed that the screening performance of NIPS in twin pregnancies is slightly reduced compared to that in singleton pregnancies, and that the false-positive and false-negative rates may be increased. Pregnant women were also told that NIPS has the potential for detecting other abnormalities, such as CNV and rare autosomal trisomies, which are indicated in the supplemental reports. If NIPS indicates that the pregnancy was high risk, further invasive prenatal diagnosis methods were required to confirm this result. Finally, the women were informed of potential situations requiring a blood redraw, such as low fetal fraction or sequencing failure. Low fetal fraction was defined as a fetal fraction < 4%. According to American College of Obstetricians and Gynecologists (ACOG) recommendations [21], we recommended that pregnant women have their blood sample recollected after two weeks (from the first blood draw); if the fetal fraction was ≥ 4% after NIPS was performed on the redrawn blood, we considered the fetal fraction to meet the quality requirements. If the second NIPS test still indicated a low fetal fraction, we provided the pregnant woman with a test failure report.

### NIPS detection of aneuploidies and CNVs and post-test NIPS genetic counseling

Maternal peripheral blood (8–10 mL) was collected from all pregnant women using cell-free BCT tubes (Streck, Omaha, NE, United States). The procedure of the NIPS test included plasma separation, cell-free DNA extraction (normal cell-free DNA concentrations ranged from 0.05 to 0.6 ng/µL), library construction (end filling and adapter ligation) and quantification (using the KAPA SYBR FAST qPCR kit), massive parallel sequencing on the NextSeq CN500 platform (Illumina, San Diego, CA), and criteria for reporting high-risk indications for aneuploidies and SCAs, as described in our previous study [22, 23]. CNVs of ≥ 2 Mb were reported in pregnant women using the Berry Genomics algorithm.

For positive NIPS results, pregnant women were informed regarding the PPV and false positive rate of NIPS-indicated aneuploidy; and for NIPS-indicated CNVs, pregnant women were informed of additional information according to the type of variant (loss or gain), location, size, whether it was a well-known microdeletion/microduplication syndrome, and our local laboratory data, such as PPV. Further invasive prenatal diagnosis was recommended, such as chorionic villus sampling (CVS), amniocentesis, and cordocentesis. Pregnant women with low risk pregnancies based on their NIPS results were informed that the fetus was at a low risk of the target disease, but that the target disease and other anomalies could not be completely ruled out, and were advised to continue ultrasound examinations as scheduled and to be monitored for any abnormal ultrasound findings.

### Prenatal diagnosis

Genetic prenatal diagnosis testing was based on karyotyping, along with CNV-seq or CMA [14, 24]. The cost of CNV-seq at our hospital was approximately 30% lower than CMA. This makes CNV-seq the preferred choice for prenatal diagnostic testing in pregnant women with isolated fetal USMs. For CNV-seq, genomic DNA was extracted and fragmented, and a DNA library was constructed. The DNA library was then quantified before undergoing massive parallel sequencing on a NextSeq 500 platform (Illumina, San Diego, CA). A total of 5 million raw sequencing reads with 36 base pair genomic DNA sequences were generated, and 2.8–3.2 million reads were uniquely mapped to the hg19 genomic sequence. For CMA, genomic DNA was extracted, amplified, fragmented, labeled, hybridized, and the single nucleotide polymorphism (SNP) array were CytoScan 750K Array(Thermo Fisher Scientific, Santa Clara, CA). The pathogenicity of CNVs identified by prenatal diagnosis was evaluated according to the ACMG guidelines. Mosaicism suggested by CMA or CNV-seq was confirmed using fluorescence in situ hybridization. All women were scheduled for a genetic counseling session to discuss pregnancy management options following prenatal diagnosis. Clinical follow-up assessments were performed from 3 months to 2 years following NIPS via telephone communication and by checking medical records.

### Statistical analysis

SPSS Statistics software (version 24.0; IBM SPSS, Armonk, NY, United States) was used for the statistical analysis. Comparisons between the groups were performed using the chi-square test. Statistical significance was set at *p* < 0.05.

## Results

### Overall study population

A total of 6869 pregnant women who met the inclusion criteria were included in this study, and 222 were excluded due to the following reasons: 186 were of advanced maternal age, 4 had sequencing failure, 7 had high cell-free DNA concentrations, and 25 had no clinical pregnancy outcome with high-risk NIPS results. The remaining 6647 pregnant women with isolated fetal USMs were included in the analysis, which included 6632 pregnant women who had a successful NIPS test and 15 pregnant women with low fetal fractions.

Blood samples for NIPS were collected in the second trimester at a median gestational age of 23 weeks (range 13–27 weeks). Maternal age ranged from 18 to 34 years (median, 27 years). The median fetal fraction shown by NIPS sequencing data was 13.41% (range 4.00–41.84%) in the 6632 pregnant women, and < 4.00% in the 15 pregnant women with low fetal fractions.

### Fetuses with suspected trisomies, SCAs and CNVs

A total of 96 pregnancies (1.45%) were suspected of having fetal chromosomal anomalies in the 6632 pregnant women. Of which, 37 pregnancies (38.54%) were positive for whole chromosome aneuploidies and 59 (61.46%) were positive for CNVs (Table 1). Detailed information on the confirmed CNVs is presented in Table 2.

**Table 1:** Summary of fetal chromosomal abnormalities identified by NIPS among the 6632 pregnant women

Fetal aneuploidies	High risk of NIPS (n)	Screen positive rate (%)	TP (n)	FP (n)	FPR (%)	PPV (%, 95% CI)	Remarks
Trisomy	14	0.21^a^	8^c^	6	0.09	57.14 (29.65–81.19)	
T21	6	0.09	4	2	0.03	66.67 (24.11–94.00)	
T18	5	0.08	4	1	0.02	80.00 (29.88–98.95)	
T13	3	0.05	0	3	0.05	–	Confirmed 1 P CNVs^e^
SCA	23	0.35^b^	7^d^	16	0.24	30.43 (14.06–53.01)	
45,X	12	0.18	2	10	0.15	16.67 (2.94–49.12)	
47,XXX	5	0.08	0	5	0.08	–	
47,XYY	3	0.05	3	0	0.00	100.00 (31.00–100.00)	
47,XXY	3	0.05	2	1	0.02	66.67 (12.53–98.23)	
CNVs	59	0.89	21	38	0.57	35.59 (23.87–49.20)	Confirmed 5 P CNVs^f^

*NIPS* noninvasive prenatal screening, *T21* trisomy 21, *T18* trisomy 18, *T13* trisomy 13, *SCA* sex chromosome aneuploidy, *CNVvs* copy number variants, *P* pathogenic, *TP* true positive, *FP* false positive, *FPR* false positive rate, *FN* false negative, *PPV* positive predictive value

^a^Screen positive rate: high-risk trisomy cases vs. high-risk CNVs cases, *p* < 0.001

^b^Screen positive rate: high-risk SCA cases vs. high-risk CNVs cases, *p* < 0.001

^c^True positive rate: true positive trisomy vs. true positive CNVs, *p* = 0.016

^d^True positive rate: true positive SCA vs. true positive CNVs, *p* = 0.008

^e^Confirmed one P CNV: non-concordant with positive NIPS (delXp21.1 (0.32 Mb))

^f^Confirmed five P CNVs: two non-concordant with positive NIPS (del15q11.2 (0.36 Mb) and del16p11.2 (0.60Mb)), two concordant with positive NIPS (positive), and one with negative NIPS (false negative, del2q37.3 (4.29 Mb))

**Table 2:** Details of confirmed fetal CNVs with positive and negative NIPS results

Sample number	Maternal age	Gestational age-weeks	USM	Maternal serum screening	hg19 co-ordinates (NIPS)	Type	Size	Z-score of CNV	Confirmatory method	Confirmatory results	ACMG Classification	Inherited or de novo	Agreement with NIPS	Outcomes
14	31	23 ^+4^	EIF	Low-risk	trisomy13	trisomy	–	–	CNV-seq	delXp21.1 (0.32 Mb)	P	NA	Discordant	TOP
38	32	22 ^+4^	EIF	Low-risk	chr13:51500000–55999999	Dup	4.5 Mb	2.25	CNV-seq	del15q11.2 (0.36 Mb)	P	NA	Discordant	TOP
39	30	17 ^+4^	EIF	NA	chr16:15500000–17999999	Del	2.5 Mb	− 9.22	CNV-seq	del16p13.11p12.3 (2.66 Mb)	P	NA	Concordant	TOP
40	29	23 ^+4^	EIF	Low-risk	chr16:15500000–18499999	Dup	3.0 Mb	7.14	CMA	del16p13.11p12.3	P	NA	Concordant	TOP
41	29	17 ^+6^	ARSA	NA	chr10:49500000–52499999	Del	3.0 Mb	− 9.95	CNV-seq	del2q37.3 (4.29 Mb) del10q11.22q11.23 (2.57 Mb)	P/VUS	de novo/Mat	Partially concordant	Birth with defects:congenital anorectal malformation, congenital heart disease, mild ventriculomegaly
42	21	24	EIF	NA	chr5:101500000–110999999	Dup	9.5 Mb	8.96	CNV-seq	dup5q21.1q22.1 (9.11 Mb)	VUS	Mat	Concordant	TOP
43	32	23 ^+3^	EIF	Low-risk	chr13:88500000–91999999	Del	3.5 Mb	− 7.54	CNV-seq	del13q31.2q31.3 (2.54 Mb)	VUS	NA	Concordant	Neonates with no phenotypical abnormalities
44	27	25	EIF	NA	chr8:3500000–6999999	Dup	3.5 Mb	6.22	CNV-seq	dup8p23.2 (2.34 Mb)	VUS	Mat	Concordant	Neonates with no phenotypical abnormalities
45	31	22 ^+3^	EIF	Low-risk	chr3:0–2499999	Dup	2.5 Mb	7.24	CNV-seq	dup3p26.3 (1.44 Mb)	VUS	NA	Concordant	Neonates with no phenotypical abnormalities
46	25	26 ^+6^	EIF	Low-risk	chr1:100000000–105999999	Dup	6.0 Mb	2.07	CNV-seq	dup1p21.2p21.1 (7.18 Mb)	VUS	NA	Concordant	Neonates with no phenotypical abnormalities
47	24	23 ^+2^	EIF	Low-risk	chr2:78500000–80499999	Dup	2.0 Mb	8.13	CNV-seq	dup2p12 (1.68 Mb)	VUS	Mat	Concordant	Neonates with no phenotypical abnormalities
48	29	27	EIF	Low-risk	chr3:0–3499999	Dup	3.5 Mb	2.73	CMA	dup3p26.3p26.2 (3.24 Mb)	VUS	NA	Concordant	Neonates with no phenotypical abnormalities
49	27	24 ^+5^	EIF	Low-risk	chr8:1500000–3999999	Dup	2.5 Mb	4.98	CNV-seq	dup8p23.3p23.2 (2.26 Mb)	VUS	Mat	Concordant	Neonates with no phenotypical abnormalities
50	25	25 ^+1^	EIF	NA	chr9:116500000–118999999	Dup	2.5 Mb	5.33	CNV-seq	dup9q32q33.1 (1.42 Mb)	VUS	Mat	Concordant	Neonates with no phenotypical abnormalities
51	25	24 ^+2^	EIF	Low-risk	chr5:124500000–126499999	Dup	2.0 Mb	6.45	CNV-seq	dup5q23.2q23.2 (1.25 Mb)	VUS	NA	Concordant	Neonates with no phenotypical abnormalities
52	23	21	EIF	Low-risk	chr4:188500000–190999999 chr9:120000000–124499999	Del, Del	2.5 Mb,4.5 Mb	− 5.65, − 6.42	CMA	del4q35.2, del9q33.1	VUS	NA	Concordant	Neonates with no phenotypical abnormalities
53	32	25 ^+5^	EIF	Low-risk	chr6:120500000–122499999	Del	2.0 Mb	− 6.94	CNV-seq	del6q22.31 (1.34 Mb)	VUS	Mat	Concordant	Neonates with no phenotypical abnormalities
54	24	25	EIF	Low-risk	chr21:44000000–46499999	Dup	2.5 Mb	5.83	CNV-seq	dup21q22.3 (2.58 Mb)	VUS	Mat	Concordant	Neonates with no phenotypical abnormalities
55	29	23 ^+6^	EIF	NA	chr21:42000000–43999999	Dup	2.0 Mb	7.47	CNV-seq	dup21q22.2q22.3 (1.56 Mb)	VUS	NA	Concordant	Neonates with no phenotypical abnormalities
56	33	18	CPCs	Low-risk	chr14:25000000–26999999	Dup	2.0 Mb	8.22	CMA	dup14q12	VUS	Mat	Concordant	Neonates with no phenotypical abnormalities
57	26	23	EIF, Mild pyelectasis	Low-risk	chr10:2000000–4499999	Dup	2.5 Mb	6.06	CNV-seq	dup10p15.3p15.2 (1.32 Mb)	VUS	Mat	Concordant	Neonates with no phenotypical abnormalities
58	34	25 ^+1^	SUA	Low-risk	chr18:0–10999999 chr18:29500000–58499999	Del, Dup	11.0 Mb, 29.0 Mb	− 1.49, 1.12	CNV-seq	dup18p11.22p11.21 (1.8 Mb), dup18q12.1q12.1 (1.32 Mb), dup18q21.33q22.1 (4.9 Mb)	VUS	NA	Partially concordant	TOP
59	29	25 ^+5^	EICF	Low-risk	chr2:105000000–106999999	Del	2.0 Mb	− 5.45	CMA	del2q12.1q12.2	VUS	Mat	Concordant	TOP
60	29	25 ^+1^	ARSA	Low-risk	–	–	–	–	CMA	del16p11.2 (0.60 Mb)	P	NA	Discordant	TOP
61	28	16 ^+6^	Mild ventriculomegaly	NA	–	–	–	–	CMA	del12q15q22 (26 Mb)	VUS	NA	Discordant	Birth with moderate ventriculomegaly
62	32	17	SUA	NA	–	–	–	–	CMA	dup5q21.3 (1.43 Mb)	VUS	NA	Discordant	Birth with mild ventriculomegaly

*NIPS* noninvasive prenatal screening, *USM* ultrasound soft marker, *CNV* copy number variant, *ACMG* American College of Medical Genetics and Genomics, *EIF* echogenic intracardiac focus, *CPCs* choroid plexus cysts, *SUA* single umbilical artery, *ARSA* aberrant right subclavian artery, *CMA* Chromosomal microarray, *P* pathogenic, *VUS* variant of uncertain significance, *Mat* maternal, *TOP* termination of pregnancy, *NA* not applicable

Of the 37 NIPS-positive fetuses, 14 fetuses were at high risk for T21 (n = 6), T18 (n = 5), and T13 (n = 3). Of them, two cases were confirmed as false positives for T21, one for T18, and three for T13 (one case was confirmed as P CNV, as shown in Table 2, case 14), yielding PPVs of 66.67%, 80.00%, and 0%, and false positive rates of 0.03%, 0.02%, and 0.05%, respectively. There were 23 fetuses at high risk for SCAs, including 12 (52.17%) with suspected 45,X, five (21.74%) with 47,XXX, three (13.04%) with 47,XYY, and three (13.04%) with 47,XXY. Furthermore, of the 23 fetuses, 16 cases were confirmed as false positive, including 10 of 12 incorrectly identified as 45,X, 5 of 5 as 47,XXX, and 1 of 3 as 47,XXY, yielding individual PPVs of 16.67%, 0%, 100.00%, and 66.67%. The total PPV of NIPS for SCA was 30.43% (95% confidence interval [CI] 14.06–53.01), and the false positive rate of NIPS for SCA was 0.24%. No false negative aneuploidy cases were observed in our study. For aneuploidies, the sensitivity of NIPS was 100.00%. Detailed information on the confirmed aneuploidies is presented in Additional file 1: Table 1.

Of the 59 positive NIPS results for CNVs, 21 cases were confirmed as true positive, including two cases with P CNVs and 19 cases with variant of uncertain significance (VUS) CNVs (one case was confirmed a true positive of VUS CNV and a false negative of P CNV, shown in Table 2, case 41), and 38 cases were confirmed as false positive, yielding a PPV of 35.59% (95% CI 23.87–49.20) and false positive rate of 0.57%. One case with P CNV was confirmed as a false negative of CNV, which was discordant with the NIPS results (Table 2, case 38).

The screening positive rate of CNVs was significantly higher than that in trisomy (0.89 vs 0.21, *p* < 0.001) and SCAs (0.89 vs 0.35,* p* < 0.001), and the true positive rate of CNVs was significantly higher than that in trisomy (0.32 vs 0.12, *p* = 0.016) and SCAs (0.32 vs 0.11, *p* = 0.008).

### Subgroup analysis of the different types of soft markers in the 6632 pregnant women

The types of soft markers and chromosomal abnormalities in different groups are listed in Table 3. EIF was the most common USM, present in 81.71% of cases (5419 of 6632), followed by CPC in 9.12% of cases (605 of 6632), and multiple soft markers in 2.90% of cases (192 of 6632). One case of T21 was detected in a fetus with EIF, one in a fetus with echogenic bowel, and two in fetuses with absent or hypoplastic nasal bone. Three cases of T18 were detected in fetuses with CPCs, and one in a fetus with SUA. Seven cases of SCA were detected in fetuses with EIF. The PPV for the whole chromosome aneuploidies was 40.54 (95% CI 25.20–57.81), with a range of 27.59–100.00% in the EIF, SUA, CPCs, echogenic bowel and absent or hypoplastic nasal bone groups. The PPV for the whole chromosome aneuploidy was significantly lower in fetuses with EIF (27.59% vs. 87.50%, *p* = 0.008) than in the other groups.

**Table 3:** PPV of NIPS for chromosomal abnormalities among the 6632 fetuses with soft markers

Ultrasound category	N (%)	Aneuploidy						CNVs				
		High risk of NIPS	True positive			PPV (%, 95% CI)	*p*	High risk of NIPS	True positive		PPV (%, 95% CI)	*p*
			T21	T18	SCA				P	VUS		
Multiple soft markers	192 (2.90)	0				–		3		1	33.33 (1.76–87.47)	1.000
EIF	5419 (81.71)	29	1		7	27.59 (13.45–47.49)	0.008^a^	50	2	15	34.00 (21.59–48.86)	0.823
Mild ventriculomegaly	7 (0.11)	0				–		0			0	
CPCs	605 (9.12)	3		3		100.00 (31.00–100.00)	0.115	3		1	33.33 (1.77–87.47)	1.000
Echogenic bowel	24 (0.36)	1	1			100.00 (5.46–100.00)	0.405	0			0	
Mild pyelectasis	154 (2.32)	0				–		0			0	
SUA	140 (2.11)	2		1		50.00 (2.67–97.33)	1.000	1		1	100.00 (5.46–100.00)	0.356
Absent or hypoplastic nasal bone	63 (0.95)	2	2			100.00 (19.79–100.00)	0.158	1			0	
ARSA	28 (0.42)	0				–		1		1^b^	100.00 (5.46–100.00)	0.356
Total (n)	6632	37	4	4	7	40.54 (25.20–57.81)		59	2	19	35.59 (23.87–49.20)	

*EIF* echogenic intracardiac focus, *CPCs* choroid plexus cysts, *SUA* single umbilical artery, *ARSA* aberrant right subclavian artery, *NIPS* noninvasive prenatal screening, *T21* trisomy 21, *T18* trisomy 18, *SCA* sex chromosome aneuploidy, *CNVs* copy number variants, *P* pathogenic, *VUS* variant of uncertain significance, *PPV* positive predictive value, *CI* confidence interval

^a^The PPV of aneuploidies was significantly lower in fetuses with EIF than in other groups (*p* = 0.008)

^b^In Case 41 (Table 2), the prenatal diagnosis confirmed one non-concordant P CNV and one concordant VUS

The P CNV 16p13.11 deletion was detected in two fetuses with EIF, the 15q11.2 deletion in one fetus with EIF, the 2q37.3 deletion in one fetus with ARSA, and the Xp21.1 deletion was identified in one fetus with EIF, of which the NIPS result was positive for T13(cases 14). NIPS identified two of 5 P CNV with positive NIPS results, and three cases were confirmed as false negatives for P CNV (Table 2, cases 14, 38 and 41). The pathogenicity ratings of the other fetal CNVs were VUS. The PPV of NIPS for CNVs detection ranged from 33.33 to 100.00% for the multiple soft markers, CPCs, EIF, SUA, and ARSA.

We evaluated the concordance of CNVs detected using NIPS and fetal confirmatory tests; two cases were classified as discordant (Table 2, cases 14 and 38) and two cases were classified as partially concordant (Table 2, cases 41 and 58). The two discordant cases were P CNVs in fetuses with EIF with sizes of only 0.32 Mb and 0.36 Mb. Among the partially concordant cases in fetuses with ARSA (case 41), NIPS was positive for the 10q11.22q11.23 deletion (3 Mb), whereas the neonatus was positive for the 10q11.22q11.23 deletion (2.57 Mb, inherited from the mother) and the 2q37.3 deletion (4.29 Mb, de novo) using postpartum diagnosis test. The 2q37.3 deletion is a P CNV that involves the 2q37.3 terminal region (including the HDAC4 gene), and the main clinical manifestations in the neonatus were congenital anorectal malformation, congenital heart disease, and mild ventriculomegaly. In the second partially concordant case in fetuses with SUA (case 58), NIPS was positive for 18p11.32-p11.21 deletion (11 Mb) and 18q12.1-q21.32 duplication (29 Mb), whereas the prenatal diagnosis confirmed 18p11.22p11.21 duplication (1.8 Mb), 18q12.1q12.1 duplication (1.32 Mb), and 18q21.33q22.1 duplication (4.9 Mb).

### Follow-up of the pregnant women with low fetal fractions

The follow-up information on the 15 pregnant women with low fetal fractions is shown in Additional file 1: Table 1 (cases 63–77). Of the 15 pregnant women, 4 were twins. Six pregnant women (including two twins) underwent amniocentesis, and no chromosomal abnormalities were identified. Nine pregnant women did not undergo invasive prenatal diagnosis. For the follow-up pregnancy outcomes, one child (case 64) had a birth defect (a congenital tracheoesophageal fistula which was surgically treated) and no prenatal or postnatal genetic tests were performed, one pregnant woman (case 70) suffered miscarriage after amniocentesis (CMA, negative), and the remaining children were evaluated by pediatricians who identified no fetal phenotypic or developmental abnormalities. Case 70 was a G2P0 woman whose NIPS indicated a low fetal fraction during her first pregnancy. Subsequent ultrasound examination indicated that the fetus had hypoplastic left heart syndrome (mitral atresia, aortic atresia with severe aortic dysplasia), ventricular septal defect, and venous catheter deficiency. Prenatal diagnosis indicated that the fetus had 22q11.2 deletion syndrome (Velocardiofacial/Di George syndrome).

### The prevalence of chromosomal aberrations in different soft marker groups

The clinical follow-up data of the 6551 pregnancies with low risk NIPS results (n = 6536) and low fetal fractions (n = 15) are shown in Additional file 2: Table 2. A total of 5947 neonates showed no phenotypic abnormalities, accounting for 90.78% of the population. Meanwhile, there were 55 abnormal cases, including 45 birth defects, seven pregnancy termination, two intrauterine fetal demise and one miscarriage after amniocentesis. Notably, one case was detected with a 16p11.2 recurrent deletion in the fetuses with ARSA, which was confirmed as a false negative of NIPS for P CNV (Table 2, case 60). Moreover, one case with 12q15q22 deletion (26 Mb, VUS) and one case with 5q21.3 duplication (1.43 Mb, VUS) and were detected (Table 2, cases 61 and 62).

The prevalence rates of aneuploidies and P CNVs in the different soft marker groups are presented in Table 4. The overall prevalence of aneuploidies and P CNVs in fetuses with USM was 0.32% (21 of 6647), which comprised 71.43% (15 of 21) with aneuploidies and 28.47% (6 of 21) with P CNVs.

**Table 4:** Prevalence rates of pathogenic chromosomal aberrations in different soft marker groups (n = 6647)

Ultrasound category	N	Chromosomal aberrations	*p* value	Aneuploidies	*p* value	P CNVs	*p* value
Multiple soft markers	192	0 (0.00)	1.000	0 (0.00)	–	0 (0.00)	–
EIF	5429	12 (0.22)	0.008	8 (0.15)	0.012	4 (0.07)	0.672
Mild ventriculomegaly	7	0 (0.00)	1.000	0 (0.00)	–	0 (0.00)	–
CPCs	609	3 (0.50)	0.663	3 (0.50)	0.313	0 (0.00)	–
Echogenic bowel	24	1 (4.17)	0.073	1 (4.17)	0.053	0 (0.00)	–
Mild pyelectasis	154	0 (0.00)	1.000	0 (0.00)	–	0 (0.00)	–
SUA	141	1 (0.71)	0.363	1 (0.71)	0.275	0 (0.00)	–
Absent or hypoplastic nasal bone	63	2 (3.17)	0.017	2 (3.17)	0.009	0 (0.00)	–
ARSA	28	2 (7.14)	0.003	0 (0.00)	–	2 (7.14)	< 0.001
Total (n)	6647	21 (0.32)		15 (0.23)		6 (0.09)	

*EIF* echogenic intracardiac focus, *CPCs* choroid plexus cysts, *SUA* single umbilical artery, *ARSA* aberrant right subclavian artery, *CNVs* copy number variants

The incidence of aneuploidies was significantly higher in fetuses with absent or hypoplastic nasal bone (3.17% vs. 0.20%, *p* = 0.009) than in the other groups. However, the incidence of aneuploidies was significantly lower in fetuses with EIF (0.15% vs. 0.57%, *p* = 0.012) than in the other groups and the incidence of P CNVs was significantly higher in fetuses with ARSA (7.14% vs. 0.06%, *p* < 0.001) than in the other groups.

## Discussion

This retrospective study determined the performance of NIPS in the detection of common aneuploidies and CNVs in fetuses with USMs in pregnant women not of AMA. Although NIPS for CNVs has been increasingly used in clinical practice, a more in-depth exploration of its accuracy and clinical utility in fetuses with USMs is needed. CNVs were the most common positive NIPS results, present in 0.89% of gravidas, followed by SCAs in 0.35% of cases, and trisomies in 0.21% of cases.

Previous prenatal diagnosis studies [14, 24] have shown that the incidence of chromosomal aberrations ranges from 2.94 to 4.34% in fetuses with USM, whereas our study showed that the total incidence of aneuploidies was 0.23% and ranged from 0 to 4.17% in different soft marker groups, and the incidence of P CNVs was 0.09%, ranging from 0 to 7.14% in different soft marker groups. This may be because the population included in our study was predominantly young (below the age of 35 years old at the expected date of confinement), while there were 9.37% pregnant women of an AMA in previous studies [14]. According to the relevant regulations (Article 20 of the Measures for the Implementation of Law of the Peoples Republic of China on Maternal and Infant Health Care (promulgated on June 20, 2001)), prenatal diagnosis is often recommended for pregnant women in China who are at high risk based on maternal serum screening [25, 26], AMA and fetuses with structural abnormalities, therefore, the risk of potential chromosomal aberrations was higher among the pregnant women included in those studies [14, 24]. In addition, the proportion of various types of USM in the study population was also different; EIF accounted for 23.72% in a previous study [14], whereas in our study, EIF was the most frequent USM (81.68%). We have previously reported [25] that the incidence and PPV of NIPS for aneuploidy in fetuses with EIF were significantly low, which was confirmed here.

Based on its performance on our cohort, NIPS retained very high sensitivity (100%) for the detection of aneuploidies; however, the PPV of NIPS for aneuploidies varied among different USMs. Our study showed that the highest PPV of NIPS for aneuploidies was found in fetuses with echogenic bowel (100%), CPCs (100%), and absent or hypoplastic nasal bone (100%), followed by those with SUA (50.00%). All SCAs were found in pregnant women with fetal EIF, and three T18 were found in pregnant women with fetal CPCs. Echogenic bowel and absent or hypoplastic nasal bone were indicative of second-trimester markers for T21, consistent with previous findings [3, 4, 27]. SUA and CPC in the second trimester of pregnancy were associated with T18, consistent with previous findings [7, 11].

CNVs have been detected in pregnant women with fetal USMs, suggesting that more attention should be paid to CNVs in pregnant women with fetal USMs, especially P CNVs [8, 15, 28–31]. In our study cohort, CNVs were more common than aneuploidies. The number of P CNVs was greater than that of fetuses with T21 and the highest incidence of P CNVs was observed in fetuses with ARSA (7.14%). We determined 2q37.3 deletion and 16p11.2 deletion in two fetuses with ARSA, whereas the most common P CNV reported in previous studies [8, 31] was a 22q11 deletion. The overall PPVs of NIPS for CNVs was 35.59% (95% CI 23.87–49.20), which were considered moderate and consistent with the results of Raymond et al.[32].

The highest PPV of NIPS for CNVs was found in fetuses with ARSA (100%) and SUA (100%), followed by those with EIF (34.00%). Although NIPS can reliably detect CNVs above 5 Mb, the clinically relevant P CNVs remain insignificant [33, 34] and NIPS may miss a meaningful CNV. In our study population, only two of six P CNVs were successfully identified using NIPS, while four of the six P CNVs were missed. Of which, three were below the resolution limit of the Berry Genomics NIPS platform (2 Mb). Although NIPS demonstrated high sensitivity for detecting common aneuploidies, it exhibited limitations in identifying P CNVs in pregnant women < 35 years old at the expected date of confinement with isolated USMs.

Although screening for CNVs using NIPS is not recommended by the American College of Obstetricians and Gynecologists [21], screening for 22q11.2 deletion syndrome is recommended by the ACMG [18]. The International Society for Prenatal Diagnosis [35] has also emphasized that clinicians must recognize that screening for genome-wide CNVs is not equivalent to screening for all P CNVs. At present, in China, screening of CNVs in pregnant women with fetal USMs depends on the cognition and counseling level of clinical genetic counselors, as well as the willingness of pregnant women, which also brings challenges and requirements for genetic counseling. It is also noteworthy to consider to the reasons for false positive and false negative NIPS results while screening for CNVs. The reason for false positive results of NIPS for CNVs are associated with maternal microduplication, confined placental mosaicism, organ transplantation, cancer, and maternal microdeletion may also lead to false negatives of NIPS for CNVs [36–38].

Low fetal fraction increase the risk of chromosomal defects and adverse obstetric outcomes [39, 40]. We usually recommend that pregnant women have their blood sample redrawn according to the ACOG recommendations [21]. Studies have also shown that the fetal fraction tends to increase with increasing gestational age [41], but some pregnant women still exhibit a low fetal fraction after their blood is redrawn. Our follow-up study also found that the proportion of adverse pregnancy outcomes among these pregnant women was increased, so following these women is critical.

### Strengths and limitations

This study has some limitations. First, the proportion of pregnant women lost to follow-up was 8.23% (539/6551) in our study population. It is difficult to precisely assess the sensitivity and specificity of NIPS for detecting CNVs as the clinical outcomes were unclear. Concurrently, during follow-up, we found 45 fetuses with birth defects, of which 43 did not undergo postpartum CMA, preventing us from confirming whether P CNVs were present. Second, the number of USMs in the different categories also varied. We identified only seven cases of fetal mild ventriculomegaly but more than 5000 of fetal EIF. In our study cohort, the composition of different USMs might have affected the overall prevalence rates of chromosomal aberrations as the potential chromosomal aberrations varied among different types of USM, possibly leading to selection bias. In future studies, we hope to accomplish the following two research objectives: (1) re-establish contact with parents of fetuses lost to follow-up to determine the presence or absence of birth defects in those children; (2) accumulate a sizeable number of cases that have both NIPS-derived clinically significant CNV as well as post-partum CMA data to assess the sensitivity and specificity of NIPS for detecting CNVs.

## Conclusion

In this study, NIPS yielded high sensitivity for the detection of common aneuploidies and SCAs and moderate PPVs for CNVs in non-AMA pregnant women with fetal USMs. Furthermore, the ability of NIPS to identify P CNVs was limited.

The incidence of aneuploidies was higher in fetuses with echogenic bowel and absent or hypoplastic nasal bone, and P CNVs were found in cases with EIF and ARSA, with significantly higher incidence in fetuses with ARSA.

In cases where NIPS indicates CNVs in pregnant women with fetal USMs, further prenatal diagnosis is strongly recommended. We do not recommend that NIPS be performed to screen for CNVs in non-AMA pregnant women with fetal USMs, especially in fetuses with ARSA.

### Supplementary Information

**Additional file 1**. Details of fetal aneuploidies detected by NIPS with validation (n = 37) and follow up of low fetal fraction cases (n = 15).

**Additional file 2**. Clinical follow-up assessment of the 6551 fetuses with low risk of NIPS (n = 6536) and low fetal fraction (n = 15).

## Data Availability

The data for this article are not publicly available because of privacy concerns. Requests to access these datasets should be directed to LHQ, hongqian.liu@163.com.

## Abbreviations

CNVs: Copy number variants
USMs: Ultrasound soft markers
NIPS: Noninvasive prenatal screening
PPV: Positive predictive value
T21: Trisomy 21
T18: Trisomy 18
T13: Trisomy 13
SCAs: Sex chromosome aneuploidies
EIF: Echogenic intracardiac focus
SUA: Single umbilical artery
ARSA: Aberrant right subclavian artery
CPCs: Choroid plexus cysts
LR: Likelihood ratio
CMA: Chromosomal microarray
CNV-seq: Copy number variant sequence
P: Pathogenic
VUS: Variant of uncertain significance
TOP: Termination of pregnancy
ACMG: American College of Medical Genetics and Genomics
ACOG: American College of Obstetricians and Gynecologists
AMA: Advanced maternal age
VTS: Vanishing twin syndrome
FGR: Fetal growth restriction
CVS: Chorionic villus sampling
SNP: Single nucleotide polymorphism
CI: Confidence interval
