# Supplementary material for: Identification of copy number variations among fetuses with isolated ultrasound soft markers in pregnant women not of advanced maternal age
Source: Orphanet J Rare Dis. 2024 Feb 10;19:56. doi: 10.1186/s13023-024-03066-4 (PMC10858470; doi:10.1186/s13023-024-03066-4)
Supplement: Supplementary file 1 — Additional file 1. Details of fetal aneuploidies detected by NIPS with validation (n = 37) and follow up of low fetal fraction cases (n = 15). [file 13023_2024_3066_MOESM1_ESM.docx]

**Supplementary Table 1.** Details of fetal aneuploidies detected by NIPS with validation (n=37) and follow up of low fetal fraction cases (n=15)

| **T21/T18/T13** | | | | | | | | | | |
| --- | --- | --- | --- | --- | --- | --- | --- | --- | --- | --- |
| Sample number | Maternal age | USM | Twin pregnancy (Y/N) | Gestational age-weeks | NIPS results | Z-score | | Diagnosis results | Agreement with  NIPS | Outcomes |
| 1 | 26 | Echogenic bowel | N | 21^+6^ | T21 | Chr21 | 6.6 | T21 | Concordant | TOP |
| 2 | 26 | EIF | N | 19^+2^ | T21 | Chr21 | 16.22 | T21 | Concordant | TOP |
| 3 | 23 | Absent or hypoplastic nasal bone | N | 14 | T21 | Chr21 | 8.28 | T21 | Concordant | TOP |
| 4 | 27 | Absent or hypoplastic nasal bone | N | 16^+3^ | T21 | Chr21 | 5.98 | T21 | Concordant | TOP |
| 5 | 23 | EIF | N | 26 | T21 | Chr21 | 3.61 | (-) | Discordant | Neonates with no phenotypical abnormalities |
| 6 | 24 | EIF | N | 25 | T21 | Chr21 | 3.84 | (-) | Discordant | Neonates with no phenotypical abnormalities |
| 7 | 32 | CPC | N | 16^+4^ | T18 | Chr18 | 5.17 | T18 | Concordant | TOP |
| 8 | 34 | SUA | N | 18 | T18 | Chr18 | 4.11 | T18 | Concordant | TOP |
| 9 | 27 | CPC | N | 17^+4^ | T18 | Chr18 | 6.4 | T18 | Concordant | TOP |
| 10 | 32 | CPC | N | 19^+1^ | T18 | Chr18 | 9.37 | T18 | Concordant | TOP |
| 11 | 29 | EIF | N | 25^+3^ | T18 | Chr18 | 6.43 | (-) | Discordant | Neonates with no phenotypical abnormalities |
| 12 | 25 | EIF | N | 24 | T13 | Chr13 | 3.74 | (-) | Discordant | Neonates with no phenotypical abnormalities |
| 13 | 33 | EIF | N | 23^+5^ | T13 | Chr13 | 3.59 | (-) | Discordant | Neonates with no phenotypical abnormalities |
| 14 | 31 | EIF | N | 23^+4^ | T13 | Chr13 | 4.29 | (-), 1 PCNV | Discordant | TOP |
| **SCAs** | | | | | | | | | | |
| Sample number | Maternal age | USM | Twin pregnancy(Y/N) | Gestational age-weeks | NIPS results | Z-score | | Diagnosis results | Agreement with  NIPS | Outcomes |
|  |  |  |  |  |  | ChrX | ChrY |  |  |  |
| 15 | 25 | EIF | N | 22^+6^ | ChrX- | -4.84 | -1.38 | Mosaic aneuploidies 45,X[20]/46,XX[80] | Concordant | TOP |
| 16 | 26 | EIF | N | 26^+5^ | ChrX- | -8.15 | -0.06 | Mosaic aneuploidies 45,X[30]/46,XX[70] | Concordant | TOP |
| 17 | 30 | EIF | N | 23^+3^ | ChrX- | -21.34 | 0.79 | (-) | Discordant | Neonates with no phenotypical abnormalities |
| 18 | 23 | EIF | N | 26^+6^ | ChrX- | -3.41 | -0.71 | (-) | Discordant | Neonates with no phenotypical abnormalities |
| 19 | 29 | SUA | N | 17^+3^ | ChrX- | -6.06 | 0.76 | (-) | Discordant | Neonates with no phenotypical abnormalities |
| 20 | 28 | EIF | N | 25^+5^ | ChrX- | -6.98 | 1.1 | (-) | Discordant | Neonates with no phenotypical abnormalities |
| 21 | 22 | EIF | N | 23^+6^ | ChrX- | -3.22 | -0.13 | (-) | Discordant | Neonates with no phenotypical abnormalities |
| 22 | 30 | EIF | N | 24^+5^ | ChrX- | -8.85 | -0.7 | (-) | Discordant | Neonates with no phenotypical abnormalities |
| 23 | 24 | EIF | N | 26 | ChrX- | -11.85 | -0.98 | (-) | Discordant | Neonates with no phenotypical abnormalities |
| 24 | 26 | EIF | N | 23^+2^ | ChrX- | -10 | 2.34 | (-) | Discordant | Neonates with no phenotypical abnormalities |
| 25 | 23 | EIF | N | 17^+1^ | ChrX- | -3.47 | 0.76 | (-) | Discordant | Neonates with no phenotypical abnormalities |
| 26 | 31 | EIF | N | 25^+2^ | ChrX- | -3.06 | -0.93 | (-) | Discordant | Neonates with no phenotypical abnormalities |
| 27 | 31 | EIF | N | 24^+2^ | ChrX+ | 3.47 | 1.67 | (-) | Discordant | Neonates with no phenotypical abnormalities |
| 28 | 25 | EIF | N | 22^+6^ | ChrX+ | 3.24 | 0.81 | (-) | Discordant | Neonates with no phenotypical abnormalities |
| 29 | 31 | EIF | N | 25^+4^ | ChrX+ | 4.01 | 0.34 | (-) | Discordant | Neonates with no phenotypical abnormalities |
| 30 | 23 | EIF | N | 23^+1^ | ChrX+ | 69.9 | 1.23 | (-) | Discordant | Neonates with no phenotypical abnormalities |
| 31 | 27 | EIF | N | 25^+3^ | ChrX+ | 3.01 | 1.58 | (-) | Discordant | Neonates with no phenotypical abnormalities |
| 32 | 33 | EIF | N | 25^+3^ | ChrY+ | 4.24 | 268.04 | 47,XYY | Concordant | TOP |
| 33 | 23 | EIF | N | 24^+5^ | ChrY+ | 3.66 | 124.05 | 47,XYY | Concordant | TOP |
| 34 | 27 | EIF | N | 23^+1^ | ChrY+ | 3.55 | 73.4 | 47,XYY | Concordant | TOP |
| 35 | 26 | EIF | N | 27 | ChrX+(Y) | 7.06 | 83.1 | 47,XXY | Concordant | TOP |
| 36 | 26 | EIF | N | 25^+1^ | ChrX+(Y) | 5.82 | 59.03 | 47,XXY | Concordant | TOP |
| 37 | 25 | EIF | N | 16^+6^ | ChrX+(Y) | 44.39 | 30.6 | (-) | Discordant | Neonates with no phenotypical abnormalities |
| **Low fetal fraction (n=15)** | | | | | | | | | | |
| Sample number | Maternal age | USM | Twin pregnancy(Y/N) | NIPS blood draw | | NIPS blood redraw | | Diagnosis method | Diagnosis results | Outcomes |
|  |  |  |  | Gestational age-weeks | Fetal fraction | Gestational age-weeks | Fetal fraction |  |  |  |
| 63 | 22 | EIF | N | 19^+1^ | 3.10 | 21^+3^ | 3.19 | NA | / | Neonates with no phenotypical abnormalities |
| 64 | 20 | SUA | N | 16^+3^ | 3.85 | 18^+3^ | 3.60 | NA | / | Congenital tracheoesophageal fistula |
| 65 | 24 | EIF | N | 16^+4^ | 3.06 | 19^+3^ | 3.62 | NA | / | Neonates with no phenotypical abnormalities |
| 66 | 25 | EIF | N | 22^+4^ | 3.66 | 25 | 3.50 | NA | / | Neonates with no phenotypical abnormalities |
| 67 | 24 | EIF | N | 23^+1^ | 3.03 | 25^+1^ | 3.43 | NA | / | Neonates with no phenotypical abnormalities |
| 68 | 20 | EIF | N | 25 | 2.98 | 27^+1^ | 3.26 | NA | / | Neonates with no phenotypical abnormalities |
| 69 | 24 | EIF | N | 23^+2^ | 3.12 | 25^+5^ | 3.99 | CNV-seq | (-) | Neonates with no phenotypical abnormalities |
| 70 | 30 | CPC | Y | 17^+2^ | 3.04 | decline | / | CMA | (-) | Miscarriage after amniocentesis |
| 71 | 32 | CPC | Y | 16^+5^ | 3.78 | 19 | 2.74 | NA | / | Neonates with no phenotypical abnormalities |
| 72 | 27 | CPC | Y | 18^+5^ | 3.55 | decline | / | NA | / | Neonates with no phenotypical abnormalities |
| 73 | 31 | EIF | N | 23^+2^ | 3.32 | 25^+2^ | 3.59 | CMA | (-) | Neonates with no phenotypical abnormalities |
| 74 | 29 | EIF | N | 26 | 3.26 | decline | / | CMA | (-) | Neonates with no phenotypical abnormalities |
| 75 | 32 | CPC | Y | 18^+4^ | 2.90 | 20^+5^ | 3.54 | CMA | (-) | Neonates with no phenotypical abnormalities |
| 76 | 28 | EIF | N | 22^+6^ | 3.70 | 25^+1^ | 2.79 | CMA | (-) | Neonates with no phenotypical abnormalities |
| 77 | 24 | EIF | N | 23^+6^ | 3.10 | 26^+4^ | 3.10 | NA | / | Neonates with no phenotypical abnormalities |

*NIPS* noninvasive prenatal screening, *USM* ultrasound soft markers, *T21* trisomy 21, *T18* trisomy 18, *SCA* sex chromosome aneuploidy, *CNV* copy-number variant, *EIF* echogenic intracardiac focus, *CP*C choroid plexus cyst, *SUA* single umbilical artery, *P* pathogenic, *VUS* variant of uncertain significance, *TOP* termination of pregnancy, *NA* not available, *CNV-seq* copy number variation sequencing, *CMA* chromosomal microarray
