# Supplementary material for: Identification of copy number variations among fetuses with isolated ultrasound soft markers in pregnant women not of advanced maternal age
Source: Orphanet J Rare Dis. 2024 Feb 10;19:56. doi: 10.1186/s13023-024-03066-4 (PMC10858470; doi:10.1186/s13023-024-03066-4)
Supplement: Supplementary file 2 — Additional file 2. Clinical follow-up assessment of the 6551 fetuses with low risk of NIPS (n = 6536) and low fetal fraction (n = 15). [file 13023_2024_3066_MOESM2_ESM.docx]

**Supplementary Table 2.** Clinical follow-up assessment of the 6551 fetuses with low risk of NIPS (n=6536) and low fetal fraction(n=15)

| Ultrasound category | Low risk of NIPS | Normal after birth | TOP/intrauterine fetal demise | Birth with defects | Confirmed CNVs | Premature delivery | Loss of follow-up |
| --- | --- | --- | --- | --- | --- | --- | --- |
| Multiple soft markers | 189 | 171 | 1 (TOP, nuchal cord) |  |  | 1 | 16 |
| EIF | 5350 | 4877 | 1 (TOP, personal reasons) 1 (intrauterine fetal demise, 24W) 1 (intrauterine fetal demise, 28W) | 2 (neonatal death) 3 (congenital heart disease) 14 (patent foramen ovale) 4 (developmental delay) 8 (hemangioma) 1 (cleft lip) 1 (hypertonia) 1 (infant ear malformations) 2 (syndactyly) |  | 6 | 428 |
| Mild ventriculomegaly | 7 | 5 |  | 1 (birth with moderate ventriculomegaly, confirmed CNVs) | del12q15q22(26Mb),VUS, Born |  | 1 |
| CPCs | 603 | 547 | 1 (TOP, ultrasound follow-up revealed structural malformations after NIPS ) 1 (miscarriage after amniocentesis) | 1 (developmental delay) 1 (hypertonia) 1 (congenital heart disease) 1 (patent foramen ovale) |  | 3 | 47 |
| Echogenic bowel | 23 | 21 |  |  |  |  | 2 |
| Mild pyelectasis | 154 | 140 |  | 1 (hydronephrosis) |  |  | 13 |
| SUA | 138 | 116 | 1 (TOP, unclear reason) 1 (TOP, oligohydramnios after NIPS) | 1 (birth with mild ventriculomegaly, confirmed CNVs) 1(congenital tracheoesophageal fistula) | dup5q21.3(1.43Mb),VUS, Born |  | 18 |
| Absent or hypoplastic nasal bone | 60 | 48 | 1 (TOP, cleft lip and palate after NIPS) | 1 (infant ear malformations) |  |  | 10 |
| ARSA | 27 | 22 | 1 (TOP, confirmed P CNVs after NIPS) |  | 16p11.2 recurrent microdeletion , P CNVs, TOP |  | 4 |
| Total (n) | 6551 | 5947 | 10 | 45 |  | 10 | 539 |

*NIPS* noninvasive prenatal screening, *CNV* copy-number variant, *TOP* termination of pregnancy, *EIF* echogenic intracardiac focus, *CPC* choroid plexus cysts, *SUA* single umbilical artery, *ARSA* aberrant right subclavian artery, *P* pathogenic, *VUS* variant of uncertain significance,
